# Does Invasion Success Reflect Superior Cognitive Ability? A Case Study of Two Congeneric Lizard Species (*Lampropholis*, Scincidae)

**DOI:** 10.1371/journal.pone.0086271

**Published:** 2014-01-24

**Authors:** Chalene N. Bezzina, Joshua J. Amiel, Richard Shine

**Affiliations:** School of Biological Sciences A08, University of Sydney, Sydney, New South Wales, Australia; Monash University, Australia

## Abstract

A species' intelligence may reliably predict its invasive potential. If this is true, then we might expect invasive species to be better at learning novel tasks than non-invasive congeners. To test this hypothesis, we exposed two sympatric species of Australian scincid lizards, *Lampropholis delicata* (invasive) and *L. guichenoti* (non-invasive) to standardized maze-learning tasks. Both species rapidly decreased the time they needed to find a food reward, but latencies were always higher for *L. delicata* than *L. guichenoti*. More detailed analysis showed that neither species actually learned the position of the food reward; they were as likely to turn the wrong way at the end of the study as at the beginning. Instead, their times decreased because they spent less time immobile in later trials; and *L. guichenoti* arrived at the reward sooner because they exhibited “freezing” (immobility) less than *L. delicata*. Hence, our data confirm that the species differ in their performance in this standardized test, but neither the decreasing time to find the reward, nor the interspecific disparity in those times, are reflective of cognitive abilities. Behavioural differences may well explain why one species is invasive and one is not, but those differences do not necessarily involve cognitive ability.

## Introduction

Species invasions are one of the largest threats to native species worldwide, but our ability to predict invasion success remains weak. To become a successful invader, a species must pass through several discrete stages of the introduction process [1]. Rather than being just a random subset of taxa, invasive species are thought to have behavioural traits that improve their chances of advancing through each of these stages [2]. Some behavioural traits may enhance a species' invasiveness across all introductory stages, whereas other traits may facilitate one stage of the invasion process but impair success at another stage. For example, “bolder” individuals may be more likely to enter a transport vector and be shipped to a new location (transport stage) but might also have a greater risk of being detected at biosecurity checkpoints (introduction stage) [2], [3]. Recently, researchers have used a variety of species-level behavioural traits to predict species' invasiveness [2].

One trait that may reliably predict species invasiveness is intelligence. We generally consider an animal to be intelligent if it is able to 1) rapidly solve novel challenges that are ecologically relevant to that species, 2) solve a single relevant challenge using multiple strategies, and 3) solve several different types of relevant challenges [4]. Once an animal arrives in a new location (i.e. after transport and introduction), it must still overcome a variety of challenges. In order to successfully establish a new population, the new arrivals must identify and avoid novel predators, locate potential mates, obtain resources, and react appropriately to unfamiliar climatic regimes. Organisms that quickly modify their behaviours to meet these challenges are more likely to survive and reproduce in their new environment and therefore, we might expect intelligence to correlate positively with invasiveness [5], [6]. Indeed, across all four classes of terrestrial vertebrates, studies that use relative brain size as a proxy for intelligence have reported that large-brained species are more successful invaders than are small-brained species [5], [7], [8]. Nonetheless, brain size is only a rough guide to intelligence, and thus these studies do not provide any direct evidence that successful invaders are better at solving novel challenges than are unsuccessful invaders. To test the hypothesis that intelligence predicts the success of species introductions, we need to specifically measure and compare the learning ability of species that have established invasive populations, compared to related but non-invasive species.

The congeneric scincid lizards *Lampropholis delicata* and *L. guichenoti* provide an ideal model system with which to test this hypothesis. These species are both small (∼35–55 mm adult snout-vent length [SVL]), oviparous (average clutch size ∼3 eggs), ground-dwelling, generalist insectivores that are broadly sympatric in suburban habitats throughout southeastern Australia [3], [9], [10]. Yet, despite these similarities, only *L. delicata* has successfully established populations outside of its native range (e.g. Lord Howe Island, The Hawaiian Islands and New Zealand), whereas *L. guichenoti* has not [3 and references therein]. However, these species co-occur in each of the areas identified as source regions for *L. delicata* introductions. Likewise, both species have been intercepted during biosecurity checks of goods entering New Zealand [3], [11], [12], suggesting that both taxa have had introduction opportunities but that only *L. delicata* has capitalised on these opportunities to become invasive.

Could differences in cognitive ability explain the apparent disparity in the ability of the two species to become established after being introduced to a new location? Certainly, behavioural traits differ between these species. For example, Chapple et al. [3] found that *L. delicata* was more exploratory than *L. guichenoti*, plausibly allowing *L. delicata* to locate critical resources and mates in novel habitats (and thus, increasing *L. delicata*'s likelihood of establishing invasive populations). However, more exploratory individuals would also encounter dangers (e.g. predators and environmental hazards) more frequently than less exploratory individuals, increasing their chances of injury and death [13]. Thus, exploratory behaviour alone seems unlikely to explain why *L. delicata* has been more successful than *L. guichenoti* at establishing populations in new locations.

Superior cognitive abilities (e.g. learning and memory) might have helped *L. delicata* to meet the challenges associated with translocation to a new habitat [5]. For example, *L. delicata* may remember the location of profitable resource patches and sensory cues associated with predators more rapidly than *L. guichenoti*, allowing *L. delicata* to maximize its chances of obtaining critical resources while reducing encounter rates with predators. Here, we test this hypothesis using a simple Y-maze with a food reward to explore whether or not *L. delicata* is able to solve a novel cognitive challenge more rapidly than does *L. guichenoti*. Because intelligence is thought to be advantageous during species introductions, we predicted *a priori* that the invasive *L. delicata* would significantly outperform its non-invasive congener *L. guichenoti* in the maze task. Such differences in cognitive ability may explain disparities in the capacity of these two species to establish populations in novel environments.

## Materials and Methods

### Ethics Statement

The University of Sydney Animal Ethics Committee approved all of the procedures described in this manuscript (approval #: L04/8-2010/3/5449). All animals were released upon completion of the study.

### Collection and Housing

We collected 16 adult *L. guichenoti* (8 adult females and 8 adult males) and 16 adult *L. delicata* (8 adult females and 8 adult males) in suburban Sydney, New South Wales, Australia. Lizards were housed in individual plastic containers (200 mm×140 mm×70 mm) lined with paper towel. Each lizard was provided with a shelter (100 mm long×230 mm diameter) and *ad libitum* access to water. We withheld food from all lizards 48 hr prior to the first maze trial in order to standardize hunger levels.

### Maze Task

**Figure 1:**
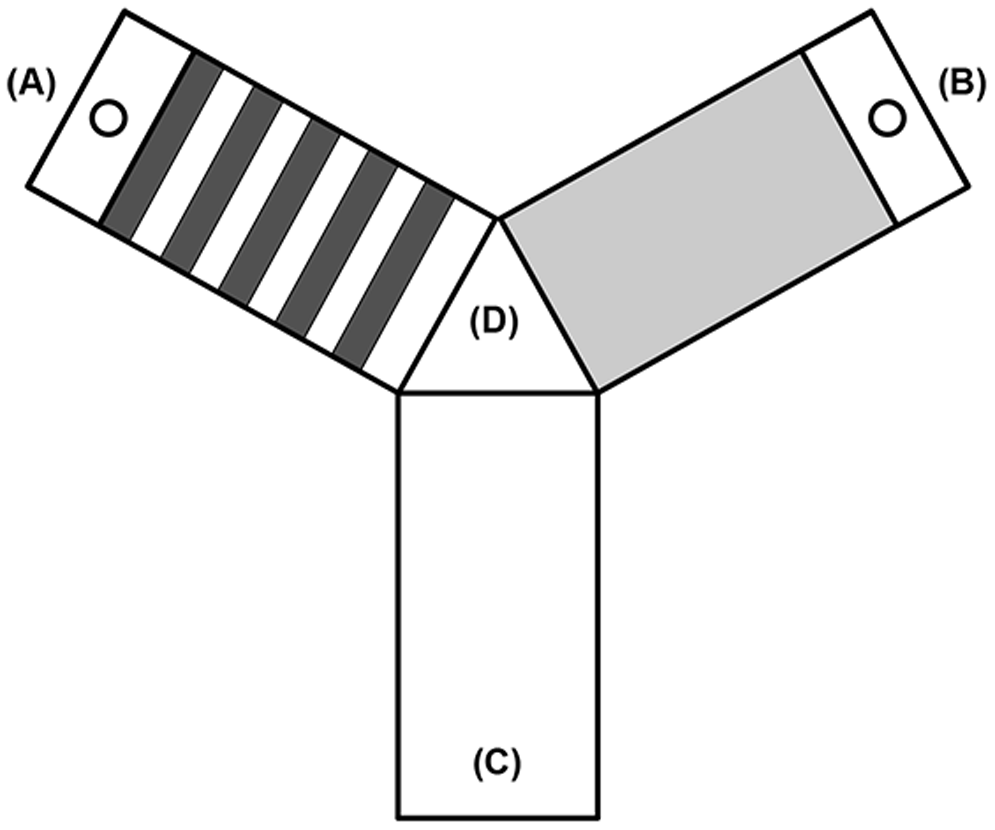
Y-mazes used to assess learning ability in *L. delicata* and *L. guichenoti*. Each maze had three arms of equal length. Two maze arms were painted with contrasting colours (orange and blue) and patterns (stripes and solids) to provide visual cues. All colour-pattern combinations were replicated and reversed in our study (four mazes total). Two arms contained feeding wells (A and B) whereas the third arm was empty and designated as the starting position for each trial (C). There was also a central decision point (D) we used to determine turning errors.

As our novel cognitive challenge, we used a simple Y-maze with a food reward to assess learning rates in *L. delicata* and *L. guichenoti*. The use of simple T- and Y-mazes to test learning ability is a standard technique in studies of reptilian cognition [14]. Mazes were constructed from opaque U-channelled electrical conduit fitted with clear plastic tops (Tripac Distribution PTY LTD, Sydney, Australia). Two arms of each maze contained a wooden platform with a single plastic feeding well. The remaining arm in each maze was empty and designated as the starting location for all trials. There was also a central decision point used to determine turning errors. As lizards use visual cues during foraging [15], the two reward-containing arms of each maze were painted with different colours (blue and orange) and patterns (striped and solid) to provide local cues. Each colour-pattern combination was replicated and reversed to account for colour bias and side preference (figure 1). Also, the mazes remained in the same location within the room used for experimental testing, providing the lizards with the opportunity to navigate the mazes using positional cues external to the maze environment.

Testing occurred over 15 days, with each lizard completing one learning trial per day. A learning trial consisted of a lizard locating a food reward (cricket, *Acheta domestica*) placed in one of the two feeding wells. For each lizard, the food reward was randomly assigned to a feeding well (left or right) prior to the start of the trial, and the location of the reward remained constant throughout the testing period. Trials began after lizards were introduced into the empty arm of the maze. We recorded the time it took for each lizard to locate the food reward (to a maximum of 30 minutes) and the direction the lizards turned after they first entered the decision point. Lizards that did not locate the food within 30 minutes were placed next to the correct feeding well and offered a cricket with forceps. All behavioural trials were run at 27°C [16] and recorded using overhead surveillance cameras (Aucom, Security, Bundoora, Australia).

### Cues and Navigation

Lizards are able to sense prey using tongue-flicking to sample chemical cues [17]. We ensured that cricket scent was present in both food wells, to eliminate scent as a long-distance direct cue to the location of the food reward (i.e. before the lizard entered one arm of the maze rather than another). We were not interested in which learning mechanisms (e.g. visual discrimination versus spatial memory) lizards used to locate their food rewards. This is because the ability to efficiently learn a novel behavioural task is likely to benefit translocated species, regardless of the mechanism they use to accomplish the task. For example, a lizard that is able to reliably locate a thermally optimal basking site may benefit by increasing the amount of time it spends at its preferred body temperature while reducing the amount of time it spends searching for basking sites in thermally sub-optimal microhabitats [18] this is true regardless of the mechanism the lizard uses to locate the site. Therefore, we provided lizards with several different types of cues (described above) that they could use to navigate the maze and locate the food reward.

### Analyses

In a maze, we recognise that an animal is capable of learning if it 1) decreases its time to locate a reward, and 2) progressively takes a more direct route to the reward over successive trials. Therefore, we used two criteria for learning: a decrease in latency to the reward across the 15 trials, and an increase in the probability of taking the most direct route to the reward across the 15 trials (described in more detail below). Because we took repeated measurements on the same individuals over time (i.e. the assumption of independence between observations was not met) and we were interested in the average responses of both species in the maze rather than subject-specific responses, we used generalized estimating equations (GEE) to determine whether or not *L. delicata* and *L. guichenoti* were capable of maze learning and whether or not the two species differed in learning rate [19].

Model 1—We were interested in whether or not both species decreased the amount of time it took them to locate the food reward across the 15 trials. We used GEE with a Gamma error distribution (log link function) and an autoregressive AR(1) working correlation matrix, to assess the relationship between mean latency to the reward (outcome variable), species and trial number (explanatory variables).

We were also interested in whether or not *L. delicata* and *L. guichenoti* behaved differently in the maze. When using latency to reach a reward as an outcome variable, consistent interspecific differences in behaviour can influence individual performance scores. For example, neophobia may cause individuals to remain motionless for long periods of time in early trials, when the maze environment is unfamiliar [20]. A more exploratory species (such as *L. delicata*) may move through a maze more readily and locate the reward faster than a less exploratory species (such as *L. guichenoti*). Such behavioural differences might lead us to infer interspecific disparities in learning abilities that do not actually exist (type I error). To control for differences in species' behaviour in the maze, we calculated the amount of time a lizard spent immobile in each trial, and included this measurement as an explanatory variable in the above GEE model.

Model 2—We were also interested in whether or not *L. delicata* and *L. guichenoti* progressively took a more direct route to the reward across the 15 trials. We assessed whether lizards turned towards the reward (i.e. took the most direct route; scored as 1) or away from the reward (i.e. deviated from the most direct route; scored as 0) when they first entered the decision point. If a lizard is learning the location of the food reward, then its probability of turning towards the reward should increase over the 15 trials. We used GEE with a Binomial error distribution (logit link function), and an AR(1) working correlation matrix to assess the relationship between direction of first turn (outcome variable), species and trial number (explanatory variables).

For both models, we chose to use AR(1) working correlation matrices because in this model the output variable depends linearly on its own previous values [21]. Similarly, we expect that a lizard's performance in the Y-maze is a function of its previous maze experience and that lizard performance will improve as the number of maze trials increases. We used a robust variance estimator, which reduces the risk of confounding effects if the empirical working correlation matrix deviates from the theoretically assumed one. Gender has the potential to influence maze learning in other taxa [22], so we initially included sex as a variable in both models. However, sex was not a significant predictor of maze performance (*P*>0.05 in all cases), so we omitted it from both models. For models 1 and 2, we included a species×trial interaction term. This interaction did not significantly predict lizard performance so we omitted it from both models. Corrected quasilikelihood under the independence model criterion (QICC) verified that the best models included only main effects. Our data did not include any missing cases. For all statistical analyses, we used SPSS v.21 and an alpha level of *P* = 0.05.

## Results

### Model 1—Latency to the Reward

**Figure 2:**
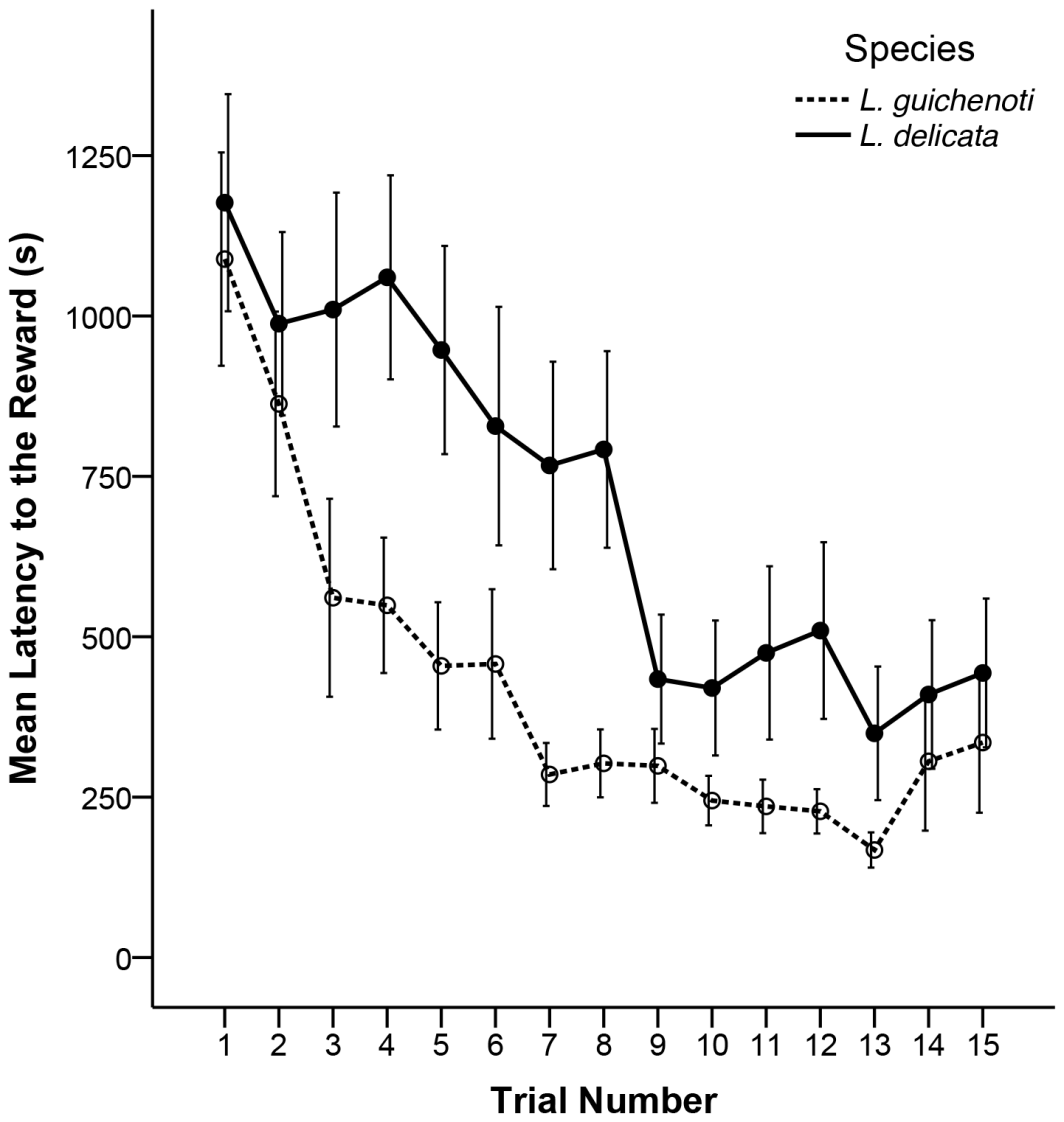
Time taken to reach food reward. Mean latency times (in seconds) for *L. delicata* (solid line) and *L. guichenoti* (broken line) to reach a food reward across 15 trials in a Y-maze. Error bars represent standard errors for each species in each trial.

**Figure 3:**
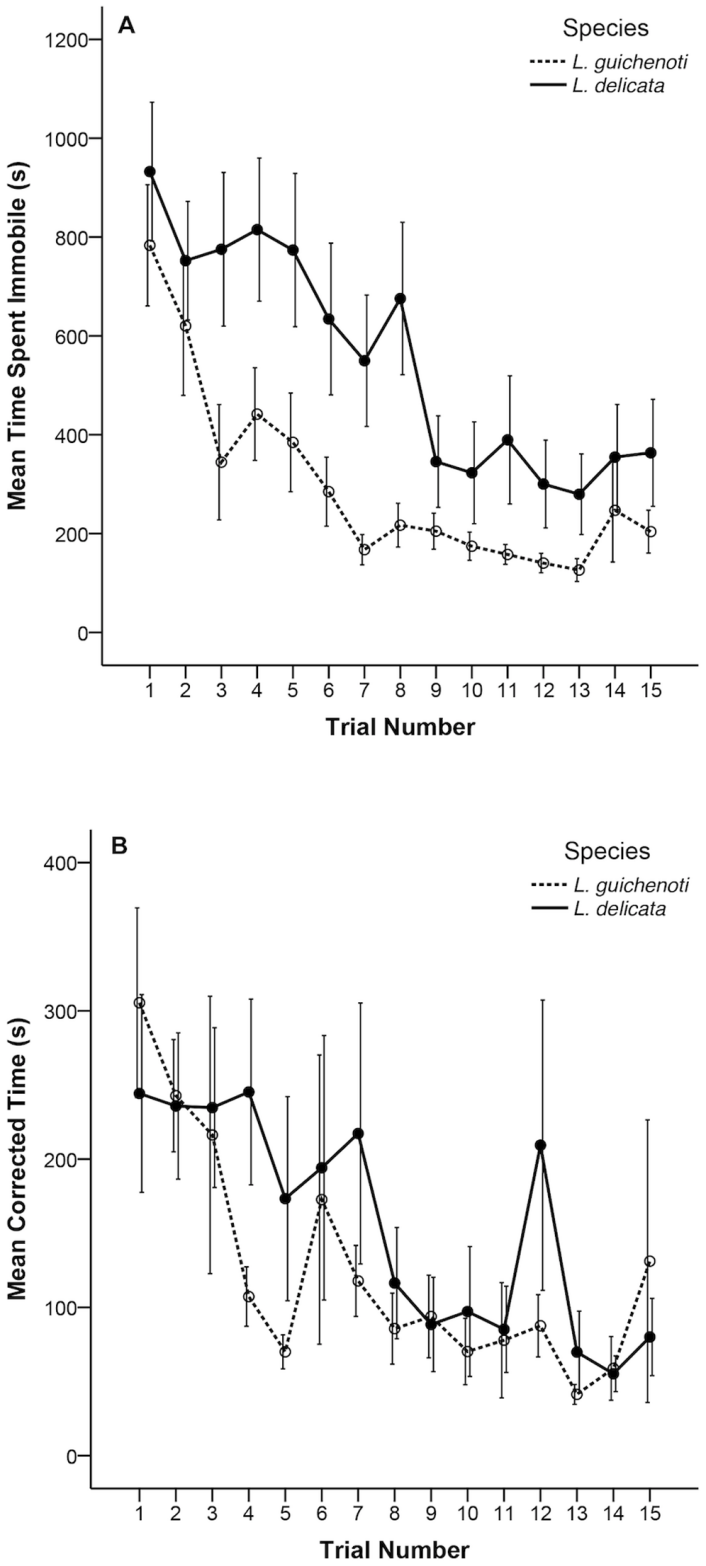
Panel A: Time spent immobile. Mean amount of time (in seconds) *L. delicata* and *L. guichenoti* spent immobile in each maze trial. Panel B: Latency times. Mean latency times (in seconds) for *L. delicata* and *L. guichenoti* corrected for the amount of time spent immobile in each trial. Solid lines represent *L. delicata* and broken lines represent *L. guichenoti*. Error bars represent standard errors for each species in each trial.

In our initial model, there was a significant effect of trial number (*P*<0.01) on latency to the reward, with the amount of time it took to locate the reward decreasing across the 15 trials in both species (figure 2). The lack of a significant species×trial interaction suggests that *L. delicata* and *L. guichenoti* decreased their mean latency times at the same rate. A significant species effect on latency to the reward (*P*<0.01) reflects the fact that *L. guichenoti* had lower mean latency times across all 15 trials than did *L. delicata* (table 1; figure 2). However, the two species also differed in the amount of time that they spent immobile (*P*<0.01), with *L. delicata* spending more time immobile than *L. guichenoti* across all 15 trials (figure 3a). Inclusion of “time spent immobile” in our model eliminated the significant difference between the two species in terms of latency to the reward (*P* = 0.17). That is, the reason that *L. delicata* took longer than *L. guichenoti* to reach the reward was simply because it spent a longer proportion of the trial immobile (table 2; figure 3b).

**Table 1:** Species differences in mean latency to the reward across 15 maze trials.

Parameter	*β*	Std. Error	95% Wald Confidence Interval		Hypthesis Test		
			Lower	Upper	Wald Chi-Square	df	*P*
Intercept	6.71	0.14	6.44	6.98	2374.17	1	<0.01
*L. delicata*	0.48	0.18	0.12	0.84	6.9	1	<0.01
Trial	0.086	0.17	−0.12	−0.053	26.40	1	<0.01
Scale	0.83						
					QICC: 367.06		

Analysis of Model 1 GEE parameter estimates based on robust variance estimates, using an AR(1) working correlation matrix, with latency to the goal as the outcome variable, and species and trial number as the explanatory variables. The QICC test of model fit is displayed in the lower right-hand corner.

**Table 2:** Species differences in time spent immobile across 15 maze trials.

Parameter	*β*	Std. Error	95% Wald Confidence Interval		Hypthesis Test		
			Lower	Upper	Wald Chi-Square	df	*P*
Intercept	5.63	0.093	5.45	5.82	3636.48	1	<0.01
*L. delicata*	0.11	0.080	−0.045	0.27	1.93	1	0.17
Trial	−0.34	0.0092	−0.052	−0.016	13.79	1	<0.01
Immobile	0.0010	0.00010	0.001	0.002	358.52	1	<0.01
Scale	0.50						
					QICC: 174.32		

Analysis of Model 1 GEE parameter estimates based on robust variance estimates, using an AR(1) working correlation matrix, with latency to the goal as the outcome variable, and species, trial number, and time spent immobile as the explanatory variables. The QICC test of model fit is displayed in the lower right-hand corner.

### Model 2—Direction of First Turn

**Figure 4:**
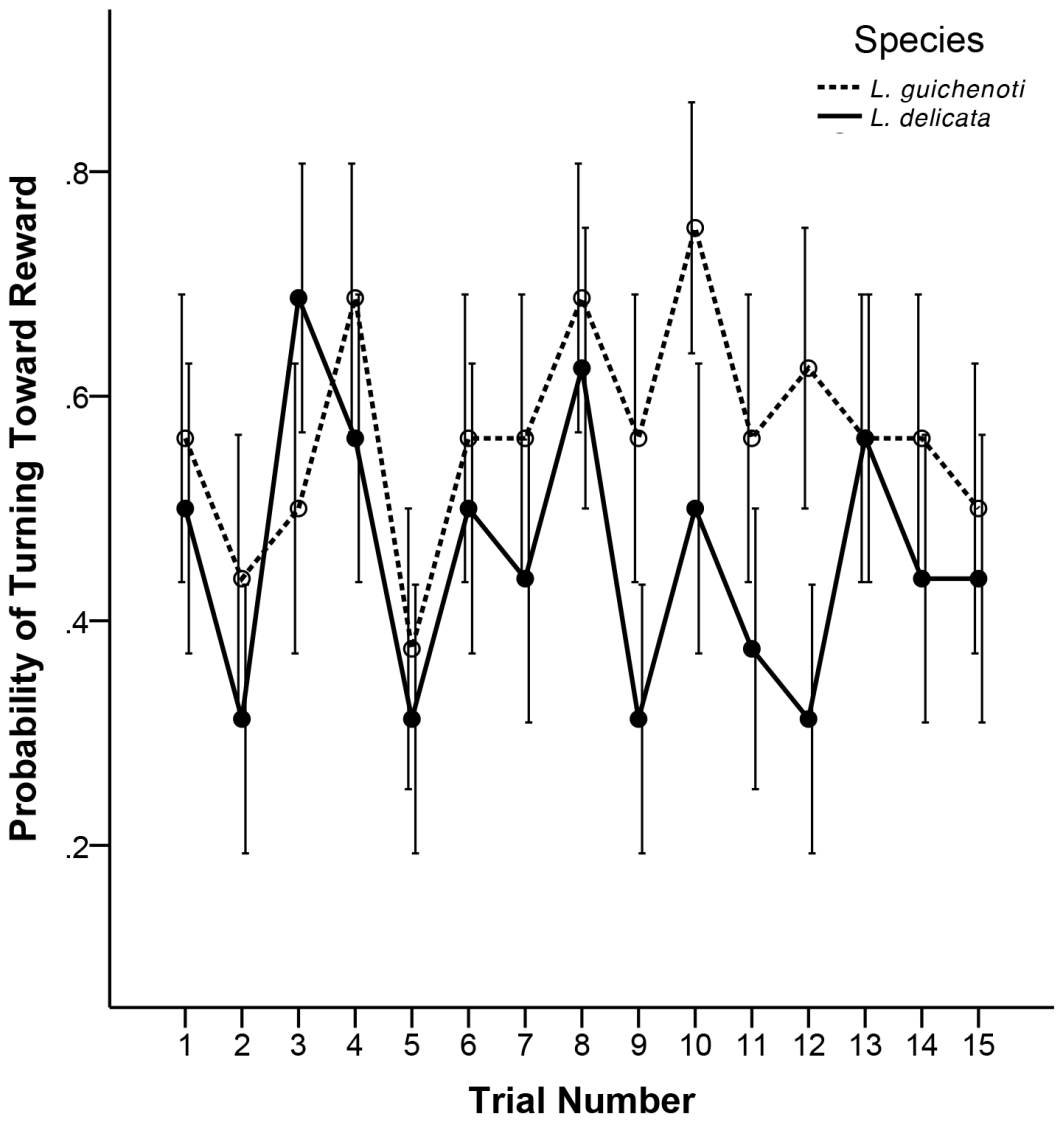
Turning probability. Probability of *L. delicata* (solid line) and *L. guichenoti* (broken line) turning towards the food reward after entering the decision point of a maze in each trial. Error bars represent standard errors for each species in each trial.

We did not find a significant effect of trial number (*P* = 0.99) or species (*P* = 0.054) on direction of first turn, suggesting that neither *L. delicata* nor *L. guichenoti* increased their probability of turning in the “correct” direction (i.e. toward the reward) as the trials progressed (table 3; figure 4).

**Table 3:** Species differences in first turn direction across 15 maze trials.

Parameter	*β*	Std. Error	95% Wald Confidence Interval		Hypthesis Test		
			Lower	Upper	Wald Chi-Square	df	*P*
Intercept	0.27	0.26	−0.23	0.77	1.09	1	0.30
*L. delicata*	−0.44	0.23	−0.88	0.0070	3.72	1	0.054
Trial	0.00	0.22	−0.043	0.043	0.00	1	0.99
Scale	1						
					QICC: 665.47		

Analysis of Model 2 GEE parameter estimates based on robust variance estimates, using an AR(1) working correlation matrix, with direction of first turn as the outcome variable, and species and trial number as the explanatory variables. The QICC test of model fit is displayed in the lower right-hand corner.

## Discussion

Our two indicators of maze learning ability were 1) a decrease in the amount of time it takes to locate the food reward, and 2) a progressively more direct route to the food reward over successive trials. Both *L. delicata* and *L. guichenoti* decreased their mean latency to the reward across 15 trials in our Y-mazes (figure 2), meeting our first criterion for maze learning. Moreover, we did not find a species×trial interaction, suggesting that both species decreased the time it took them to solve the maze at the same rate. *Lampropholis guichenoti* had lower mean latency times across all 15 trials (figure 2), suggesting that they consistently outperformed *L. delicata*; however, once we considered the time both species spent immobile (i.e. compared latency times when the lizards were actually moving), there was no significant interspecific difference in mean latencies (figure 3b).

The interspecific differences we recorded in maze behaviour (i.e. time spent immobile) may be due to habitat preferences. Not only are *L. delicata* more commonly found in vegetated rather than open areas [3], but Chapple et al. [3] reported that they spend more time hiding than *L. guichenoti* when provided with the opportunity to seek shelter in a laboratory experiment. Thus, *L. delicata* may be less “comfortable” in the open setting of the maze than are *L. guichenoti*. Modification of the maze environment might allow a more accurate measure of *L. delicata*'s ability to complete the task. We predict that inclusion of refuges or covered ledges within the maze would reduce the time that *L. delicata* spends immobile, and increase the species' overall performance - perhaps to the point that this species performs as well as does its bolder congener, *L. guichenoti*. If animals that prefer open habitats perform better in mazes, this result could have important implications for the use of mazes in intraspecific as well as interspecific comparisons of cognitive ability. Juvenile reptiles often spend more time in covered habitats than do conspecific adults [23]. Also, pregnant female reptiles often prefer more sheltered habitats than do non-pregnant females or males [24]. This ecological heterogeneity means that variables such as age, sex and reproductive status may influence the performance of individuals in a maze task. In any cognitive test, contextual variables can influence the performance of individuals [25]. Maze designs that take into account a species' preference for sheltered or open habitats may drastically improve the performance of animals in a maze and reduce the magnitude of type I errors in comparative cognition studies.

Our next goal was to determine whether or not *L. delicata* and *L. guichenoti* decreased their mean latency times by taking a more direct route to the food reward. Neither species increased their probability of turning in the correct direction toward the reward across the 15 trials, and we did not find a species effect on direction of the first turn (figure 4). This result suggests that neither *L. delicata* nor *L. guichenoti* progressively took a shorter route to the food reward across the 15 trials and thus, our second criterion for maze learning was not met. If a species steadily decreases its time to locate a reward within a maze without taking a more direct route, it suggests that the species has habituated to the maze environment and simply searches the maze more rapidly over successive trials [20]. In keeping with this interpretation, our data show a rapid decrease in the amount of time spent immobile in successive trials (figure 3a). Based on these data, neither *L. delicata* or *L. guichenoti* were capable of learning the position of a food reward within a Y-maze. Instead, both species appeared to locate the reward by performing increasingly rapid serial searches of the maze environment. However, our results do not preclude the possibility that *L. delicata* and *L. guichenoti* are capable of learning the location of a food reward within a Y-maze. Given a greater number of trials, *L. delicata* and *L. guichenoti* may have learned to reliably locate the reward.

Species that can rapidly solve novel ecological challenges are predicted to have an advantage during introduction events [6], [8]. Although neither *L. delicata* nor *L. guichenoti* learned the location of a food source within 15 trials, this result does not mean that intelligence is irrelevant to establishment success. We only tested learning ability in a single behavioural context, which is unlikely to provide an accurate representation of species' intelligence [4], [26]. Further cognitive testing using multiple experimental frameworks may reveal that *L. delicata* is capable of solving a variety of ecologically relevant challenges more efficiently than can *L. guichenoti*. Such experiments would give a more comprehensive comparison of cognition between these two species, and a more robust test of the hypothesis that intelligence facilitates invasion success.

Another factor that may influence learning ability and therefore, invasive potential, is age. As animals age, cognitive functions such as learning and memory deteriorate [27]. Indeed, using the same Y-mazes and a similar experimental design, we found that hatchling three-lined skinks (*Bassiana duperreyi*) decreased their time to locate a food reward and took a more direct route to the reward across 15 trials, providing strong evidence that *B. duperreyi* learned the location of the food reward [14]. It would be interesting to see if hatchling *L. delicata* and *L. guichenoti* display a similar capacity for learning as do hatchling *B. duperreyi*. Interspecific disparities in learning ability may also be more apparent in younger age classes. These hypotheses could be easily tested using hatchling *L. delicata* and *L. guichenoti*, and the same experimental design we used in the present study. If greater learning ability does correlate positively with establishment success, then the age of individuals at the time of introduction may be a strong predictor of invasive potential.

Finally, learning ability might not have a significant influence on establishment success at all. Rather, alternative pre-existing behavioural traits (such as aggressiveness, and habitat preference and flexibility) may predict establishment success more accurately. For example, Chapple et al. [3] suggest that relative to *L. guichenoti*, *L. delicata*'s exploratory nature and propensity to hide increases its propagule pressure during introductions, which in turn increases the probability that *L. delicata* will successfully establish in new environments. If other behavioural traits can predict establishment success more effectively than intelligence, does this not invalidate previous studies, which have suggested that relatively large brains provide translocated individuals with a selective advantage [6]–[8]? Not necessarily. A larger brain may have numerous functional consequences, including non-cognitive advantages in sensory and motor functions. Any such attribute plausibly could enhance invasion success [7]. Therefore, the putative role of intelligence as a predictor of invasion success remains unclear. In order to understand this relationship, we will need to explore cognitive disparities among successful and unsuccessful invaders in a variety of different contexts.
